# The third form electric organ discharge of electric eels

**DOI:** 10.1038/s41598-021-85715-3

**Published:** 2021-03-18

**Authors:** Jun Xu, Xiang Cui, Huiyuan Zhang

**Affiliations:** School of Electrical and Electronic Engineering, North China Electric Power University, Beinong Road 2, Huilongguan, Changping District, Beijing, 102206 China

**Keywords:** Animal behaviour, Animal physiology

## Abstract

The electric eel is a unique species that has evolved three electric organs. Since the 1950s, electric eels have generally been assumed to use these three organs to generate two forms of electric organ discharge (EOD): high-voltage EOD for predation and defense and low-voltage EOD for electrolocation and communication. However, why electric eels evolved three electric organs to generate two forms of EOD and how these three organs work together to generate these two forms of EOD have not been clear until now. Here, we present the third form of independent EOD of electric eels: middle-voltage EOD. We suggest that every form of EOD is generated by one electric organ independently and reveal the typical discharge order of the three electric organs. We also discuss hybrid EODs, which are combinations of these three independent EODs. This new finding indicates that the electric eel discharge behavior and physiology and the evolutionary purpose of the three electric organs are more complex than previously assumed. The purpose of the middle-voltage EOD still requires clarification.

## Introduction

Humans have known of the special ability of electric eels to stun prey and people since ancient times^1,2^. This special force of “electricity” has attracted the attention of scientists since the early stage of the development of science^1–3^: Williamson and Walsh^2,3^, Von Humboldt^2,3^, and Faraday^3,4^ used electric eels as electricity sources in experiments. The electricity utilized by these scientists is currently known as high-voltage electric organ discharge (EOD), which can reach at most 860 V according to recent research^5^. Biologists Hunter^6^ and Darwin^7^ dissected and studied the electric eel. Hunter^6^ reported on the electric eel electric organs in 1775 and found that these eels have two separate electric organs. Sachs^8^ found the third electric organ in 1877. Therefore, to date three pairs of electric organs of electric eels have been described in Electrophorus, and these are known as the Hunter’s organ, the main organ and the Sachs’ organ^9–13^. Coates et al.^14^ reported that electric eels can generate a second form of EOD, i.e., low-voltage EOD, in 1937. In 1958, Lissmann^15^ found that weak electric fish use low-voltage EOD for electrolocation and communication, thus revealing the function of the gymnotiform low-voltage EOD. Catania^16^ reported that electric eels emit three distinct types of EOD: (i) low-voltage pulses for sensing their environment, (ii) pairs and triplets of high-voltage EODs given off periodically while hunting in complex environments, and (iii) high-frequency volleys of high-voltage pulses during prey capture or defense. Although Catania classified EODs into three types, both type 2 and type 3 EODs are high-voltage EODs. From the voltage amplitude point of view, two types of EOD have been found: high-voltage EOD for predation and defense and low-voltage EOD for navigation and communication. Since the 1930s, electric eels have been assumed to control these three pairs of organs to generate the above two forms of EOD, with many studies related to electric eel discharge behavior and physiology being based on this assumption^1,5,10,11,16^.

However, this assumption has two problems: (1) Why did electric eels evolve three separate electric organs to generate two forms of EOD? (2) How do these three electric organs work together to generate these two forms of EOD? Problem 1 has yet to be solved, but some conflicting descriptions regarding Problem 2 exist in different papers: Traeger et al.^10,11^ and Crampton^17^ described that high-voltage EOD was generated by the main organ and the anterior portion of Hunter’s organ, while low-voltage EOD was generated by Sachs’ organ and the posterior portion of Hunter’s organ. Gotter et al.^18^, Sillar et al.^19^ and Souza et al.^20^ described that high-voltage EOD was generated by the main organ, while low-voltage EOD was generated by Sachs’ organ and Hunter’s organ. The key point is that the discharge mode and function of Hunter’s organ are not clear, even though this organ was found in 1775^6^.

By precisely measuring the voltage waveform and space distribution along an electric eel’s body, we found that electric eels generate three independent forms of EOD instead of just two, and each EOD voltage space distribution matched one electric organ’s shape and position: The low-voltage EOD space distribution matched the shape and position of Sachs’ organ. The high-voltage EOD space distribution matched the shape and position of the main organ. The third EOD form, which we define as middle-voltage EOD, has an amplitude that is approximately 2.4 times that of low-voltage EOD and 1/5 that of high-voltage EOD and a voltage space distribution along the body of an electric eel that matches the shape and position of Hunter’s organ. This observation indicates that electric eels use three pairs of electric organs to generate three forms of EOD independently. We also found some irregular EOD waveform, and they can be explained as simply combinations of low-voltage EOD, middle-voltage EOD and high-voltage EOD. This conclusion reveals that the electric eel discharge behavior and physiology are more complex than previously assumed. However, the purpose of the middle-voltage EOD and why electric eels evolved Hunter’s organ still require clarification.

## Results

### The setup of the experiments

**Figure 1:**
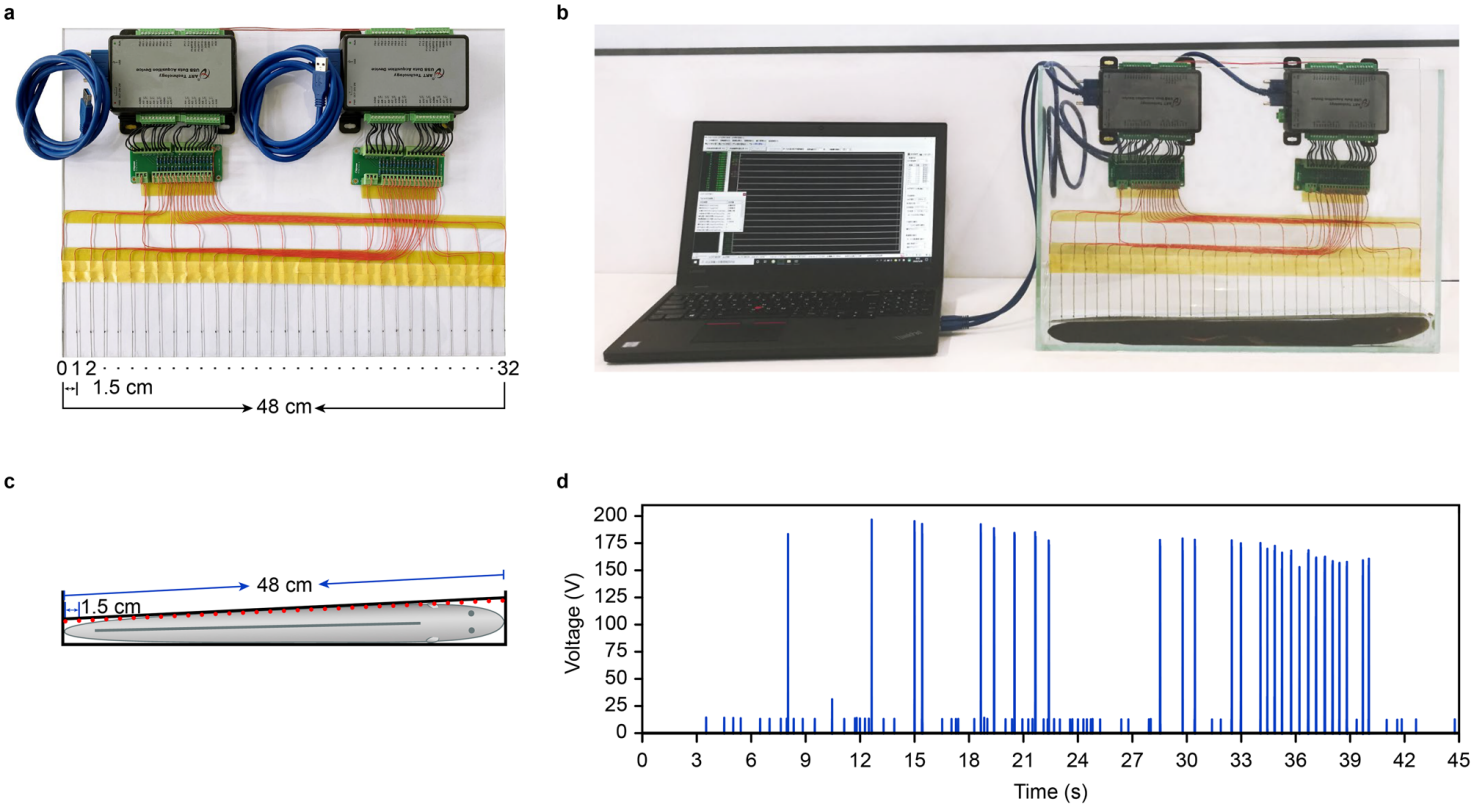
Using 32 channels AD Converter to record electric eel discharge. (**a**) Acrylic glass to which 2 × 16 channel AD converters and 33 electrodes were attached. (**b**) Measuring and recording the electric eel discharge. (**c**) Schematic of the voltage-measuring electrodes and the electric eel. (**d**) The discharge waveform recorded by electrode 32 (using electrode 0 as reference).

We experimented on a 48 cm long *Electrophorus* *electricus* and placed it into a 50 × 32 × 35 cm (L × W × H) glass tank. We then designed a 48 × 36 cm (L × H) acrylic board, which was used to mount the voltage-measuring electrodes. We employed 33 electrodes made of nickel-titanium alloy, as shown in Fig. 1a. The far-left electrode, i.e., electrode 0, works as a voltage and distance reference point: assume that its voltage is 0 V and its location is 0 cm. The other 32 electrodes, i.e., electrode 1 to electrode 32, were used to measure the voltage. The distance between two electrodes was 1.5 cm, and the total distance was equal to the electric eel length of 48 cm. The 32-channel 16-bit AD converter we used had a sample rate of 31.25 ksps per channel, and the voltage-measuring span was ± 300 V. As a result, the voltage-measuring system time resolution was 0.032 ms, voltage resolution was 9.16 mV and space resolution was 1.5 cm. We used the acrylic board to slightly push the electric eel toward the glass tank side, make the electrode 0 contacted to the tip of the tail of the eel, as shown in Fig. 1b. Fresh water was added to the glass tank to a depth of 4 cm, which just covered the electric eel’s back. The water ensured that all 33 electrodes could measure some voltage. Though a few of them may not have contacted the electric eel skin well, the water voltage measured was very similar to the skin voltage because of the short distance. Figure 1c presents the voltage-measuring system schematic.

We used a glass rod to touch the electric eel’s head to evoke discharge and a computer to record all 32 electrode voltage waveforms. Figure 1d shows the voltage waveform recorded by electrode 32. It lasted 45 s totally. The analysis below is based on the 45 s data of all 32 electrodes recorded.

### Analysis of low-voltage EOD

**Figure 2:**
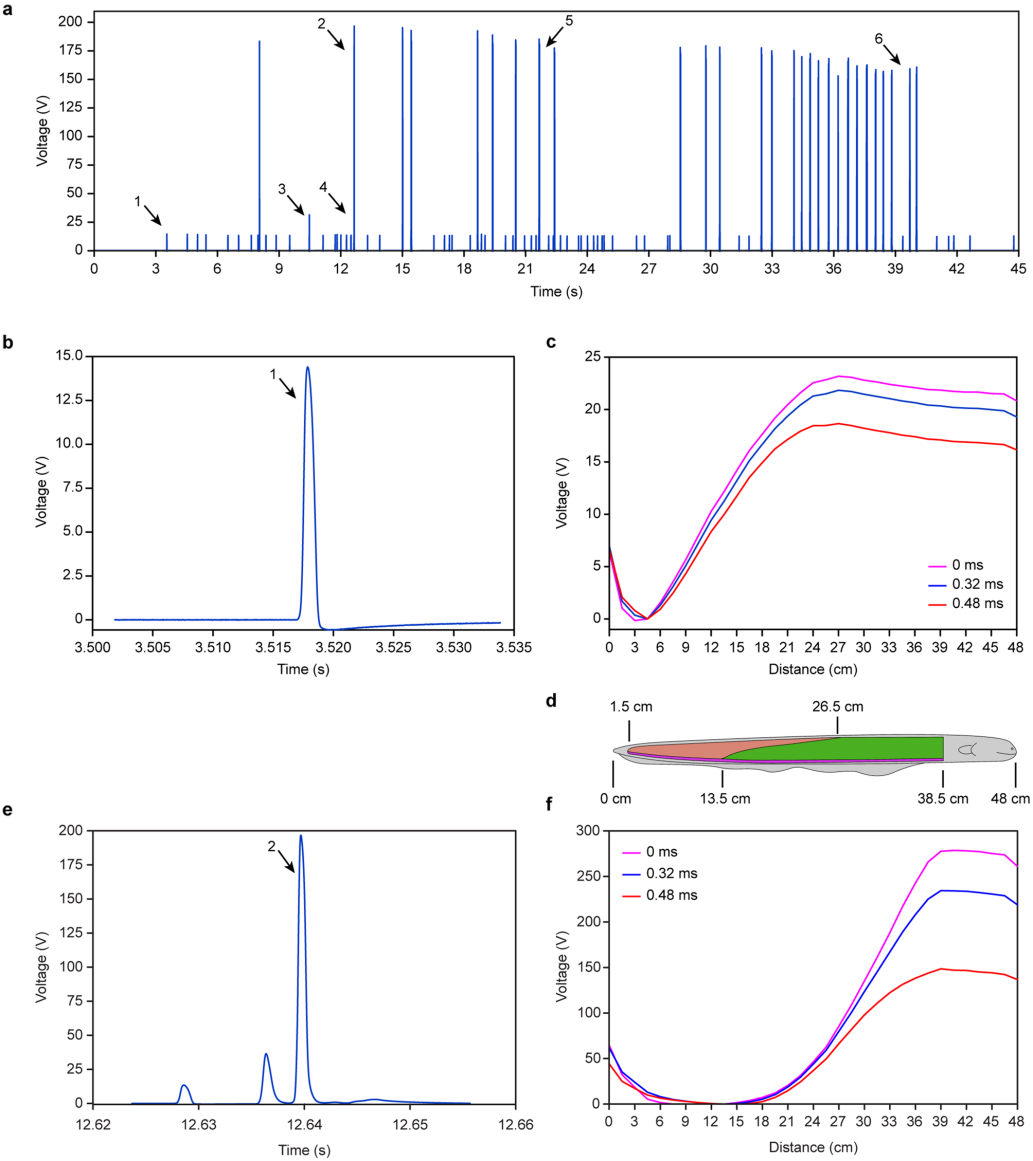
EOD voltage waveform and space distribution. (**a**) The discharge waveform recorded by electrode 32(using electrode 0 as reference) (**b**) Enlarged view of the low-voltage EOD 1. (**c**) The voltage space distribution along the electric eel body in the period of low-voltage EOD 1 (using electrode 3 as reference). (**d**) The 48 cm long electric eel and its three electric organs: the orange part shows Sachs’ organ, the green part shows the main organ, and the purple part shows Hunter’s organ. (**e**) Enlarged view of the high-voltage EOD 2(using electrode 0 as reference). (**f**) The voltage space distribution along the electric eel body in the period of high-voltage EOD 2 (using electrode 9 as reference).

We first analyzed the low-voltage EOD waveform at 3.518 s, as shown in Fig. 2a EOD 1. This EOD was also enlarged, as shown in Fig. 2b. When checking all 32 electrode voltages at the moment of the EOD 1 pulse peak, we found that the electrode 3 voltage was the lowest and less than 0, which means that electrode 3 is the negative pole of this EOD. Then, we used electrode 3 as the voltage reference point, and all 33 electrode voltages (including the 0 V of electrode 0) were subtracted from electrode 3 voltage. The results are represented in Fig. 2c by the magenta line. The horizontal axis is the distance from every electrode to the electric eel’s tail end, and the vertical axis is the voltage amplitude of every electrode relative to that of electrode 3. Clearly, the voltage of electrode 18 is the highest, reaching 23 V. According to electromagnetic field theory, the negative pole should be located near electrode 3 at the 4.5 cm ± 0.75 cm position, and the positive pole should be located near electrode 18 at 27 cm ± 0.75 cm.

The blue line is the waveform 0.32 ms after the pulse peak instant, and the red line is the waveform 0.48 ms after the pulse peak instant. From these three lines, we can find that all 33 electrode voltages reached their maximum value at the same time and dropped synchronously, which means that no voltage phase delay occurs along the electric eel’s body within our measurement accuracy. Supplementary Movie 1 demonstrates the changing process of this low-voltage EOD.

We dissected this eel. Figure 2d shows its three electric organs boundary. Compared Fig. 2c with Fig. 2d, we found that this EOD negative pole and positive pole position matched the position of Sachs’ organ. The voltage increased with distance in the 4.5–27 cm portion, but the increasing rate decreased with distance, which matched the shape of Sachs’ organ: it becomes narrower along the electric eel’s body, which means fewer electrocytes and a lower rate of voltage increase. We thus concluded that low-voltage EOD is independently generated by Sachs’ organ.

### Analysis of high-voltage EOD

We analyzed the high-voltage EOD at 12.64 s, as shown in Fig. 2a EOD 2. This EOD was also enlarged, as shown in Fig. 2e. When checking all 32 electrode voltages at EOD 2 pulse peak, we found that electrode 9 had the lowest voltage among all 32 electrodes and was less than 0, which means that electrode 9 is the negative pole of the high-voltage EOD. Then, we used electrode 9 as the voltage reference point, and all 33 electrode voltages (including the 0 V of electrode 0) were subtracted from that of electrode 9, the results of which are depicted by the magenta line in Fig. 2f. The voltage of electrode 26 was the highest, reaching 278 V. Then, the negative pole was located at 13.5 cm ± 0.75 cm, and the positive pole was located at 39 cm ± 0.75 cm. The blue line is the waveform 0.32 ms after the pulse peak instant, and the red line is the waveform 0.48 ms after the pulse peak instant. Supplementary Movie 2 demonstrates the changing process of this high-voltage EOD.

The high-voltage EOD negative pole and positive pole positions matched the main organ’s position, as shown by the green region of Fig. 2d. The voltage increased with the distance in the 13.5–39 cm portion, and the increasing rate increased with the distance also, which matches the shape of the main organ: it became wider along the electric eel’s body, which means more electrocytes and a higher rate of voltage increases. Thus, we can conclude that high-voltage EOD is independently generated by the main organ.

Compared the low-voltage EOD with the high-voltage EOD, we found that their waveforms in the time domain are very similar, except for the amplitude difference, as Fig. 2b,e show. However, a large difference in the voltage space distribution along the electric eel’s body exists, as shown in Fig. 2c,f. The negative pole and positive pole positions apparently changed. This change means that the traditional “standard” electric eel discharge voltage measurement method, which measures the voltage from the tip of the snout to the tip of the tail^5,21^, is an underrepresentation of the maximum voltage of the high-voltage EODs and low-voltage EODs, measured between the positions of the negative and positive poles. Measuring the voltage between the two ends of the electric organ will yield a more precise result.

### The third form EOD: middle-voltage EOD

**Figure 3:**
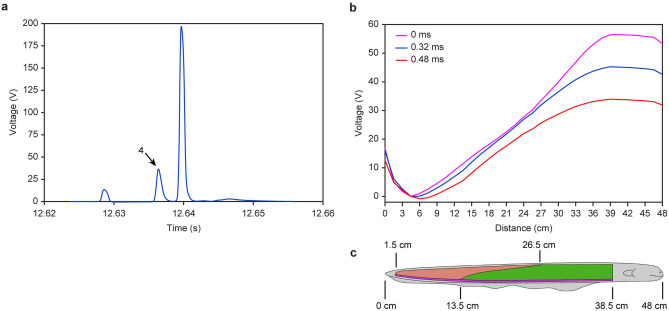
The third form EOD: middle-voltage EOD. (**a**) Enlarged view of the EOD 4 as shown in Fig. 2a (using electrode 0 as reference). (**b**) The voltage space distribution along the electric eel body in the period of middle-voltage EOD 4 (using electrode 3 as reference). (**c**) The 48 cm long electric eel and its three electric organs.

We found that some EOD voltages are different from the low-voltage EOD and high-voltage EOD, as shown in Fig. 2a (EOD 3 and EOD 4). EOD 4 is not clear in Fig. 2a but clear in Fig. 3a when enlarged. The EOD waveform is similar to the low-voltage EOD and high-voltage EOD except for the amplitude. The electrode 3 voltage is the lowest among all 32 electrodes and less than 0 at the pulse peak instant. Then, we used electrode 3 as the voltage reference point, and all 33 electrode voltages (including the 0 V of electrode 0) were subtracted from electrode 3 voltage. The results are represented in Fig. 3b by the magenta line. The voltage of electrode 26 is the highest, which means that the negative pole is located at 4.5 cm ± 0.75 cm and that the positive pole is located at 39 cm ± 0.75 cm. The blue line is the waveform 0.32 ms after the pulse peak instant, and the red line is the waveform 0.48 ms after the pulse peak instant. Supplementary Movie 3 demonstrates the changing process of this middle-voltage EOD.

The maximum voltage of the magenta line reaches 56 V, which is 2.4 times that of the low-voltage EOD and 1/5 that of the high-voltage EOD. This EOD negative pole is the same as the low-voltage EOD, but the positive pole is the same as the high-voltage EOD. This EOD voltage space distribution waveform is similar to a straight line, different from the low-voltage EOD convex waveform in Fig. 2c and the high-voltage EOD concave waveform in Fig. 2f. Thus, this EOD is a completely new one that is different from low-voltage EOD and high-voltage EOD, which we define as middle-voltage EOD.

The middle-voltage EOD negative pole and positive pole matched the position of Hunter’s organ, as shown by the purple region of Fig. 3c. The voltage increased with distance in the 4.5–39 cm portion, and similar to a straight line, the rate increase with distance was constant. This observation matched the constant width of Hunter’s organ along the electric eel’s body, from which we can conclude that the middle-voltage EOD is generated by Hunter’s organ independently.

### Analysis of irregular EOD

**Figure 4:**
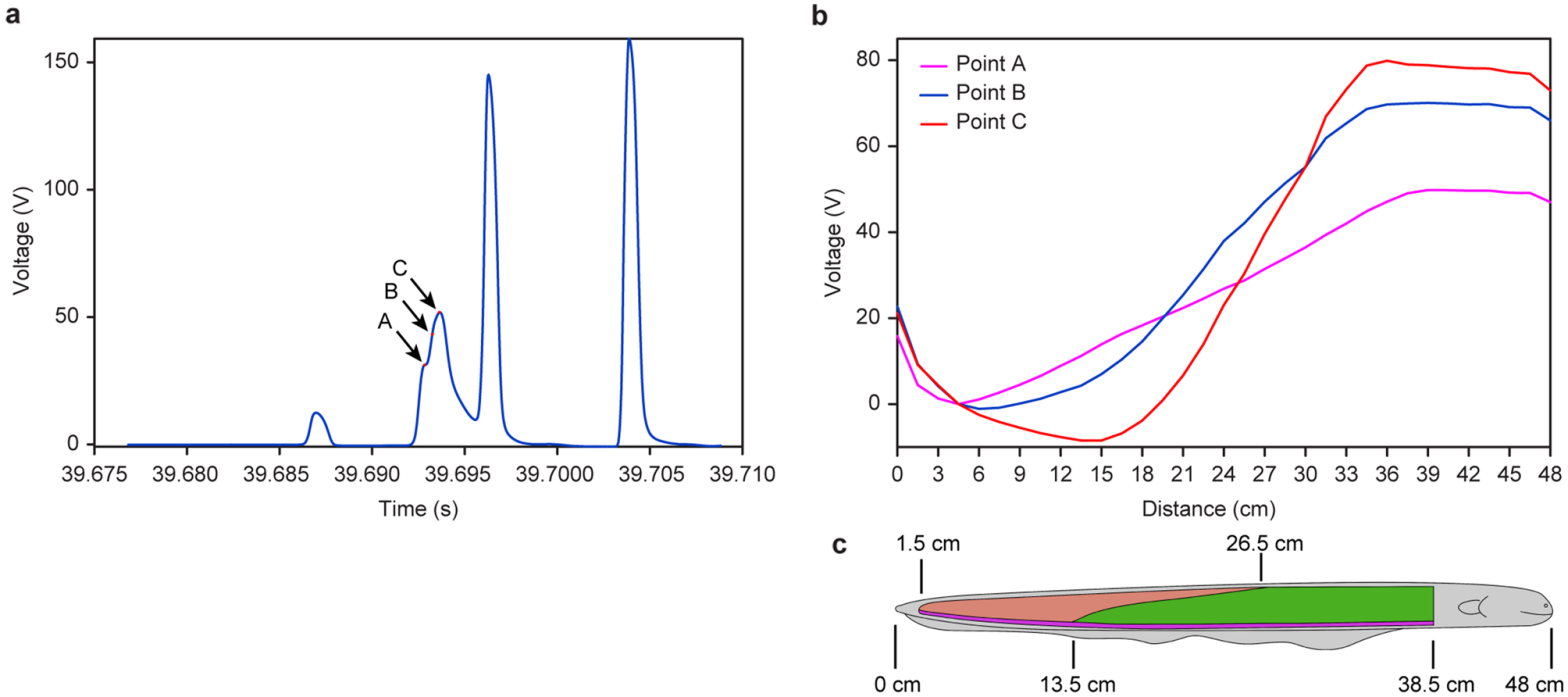
The irregular EOD. (**a**) Irregular EOD waveform, which is an enlarged view of the EOD 6 in Fig. 2a (using electrode 0 as reference). (**b**) Comparison of the three different instant voltage space distribution along the body of 48 cm long electric eel (using electrode 3 as reference) (**c**) The 48 cm long electric eel and its three electric organs.

According to circuit theory, these three EODs have different voltages; if two or three of them discharge at the same time, a loop current will flow inside the electric eel body. This current will increase the voltage of the lower EOD but decrease the voltage of the higher EOD, harming the EOD normal function. However, we found some irregular EOD waveform, they can be explained only as the discharging of two or three electric organs, as shown in Fig. 2a EOD 6, This EOD was also enlarged, as shown in Fig. 4a.

The pulse waveform in Fig. 4a at 39.693 s is not a typical electric eel EOD, its duration is longer than that of any kind of normal EOD, and the waveform increasing trend changes at point A. By analyzing the voltage space distribution at point A, point B (0.384 ms after point A) and point C (0.768 ms after point A), the results are as shown in Fig. 4b (using electrode 3 as reference). Compared with Fig. 4c, we find that the voltage space distribution at point A is a typical middle-voltage EOD but at point C is a typical high-voltage EOD. The voltage space distribution at point B seems to be an intermediate waveform. This EOD pulse can be explained as follows: The electric eel emits a middle-voltage EOD following a low-voltage EOD as the normal but launches the high-voltage EOD at the wrong time, i.e., before the middle-voltage EOD has finished. Then, the high-voltage EOD conflicts with the middle-voltage EOD, generating an internal loop current and causing high-voltage EOD “failure” (the voltage achieved was only 80 V). The electric eel launched another high-voltage EOD soon after (1.92 ms after point C) to “make up for this mistake”. Therefore, this irregular EODs can be treated as malfunctions of electric eel discharge order. Some other irregular EOD waveform that can also be explained by the combination of low-voltage EOD, middle-voltage EOD and high-voltage EOD also exist. We define low-voltage EOD, middle-voltage EOD and high-voltage EOD as basic EODs and other irregular EODs as hybrid EODs. Supplementary Movie 4 demonstrates the changing process of this irregular EOD.

## Discussion

**Figure 5:**
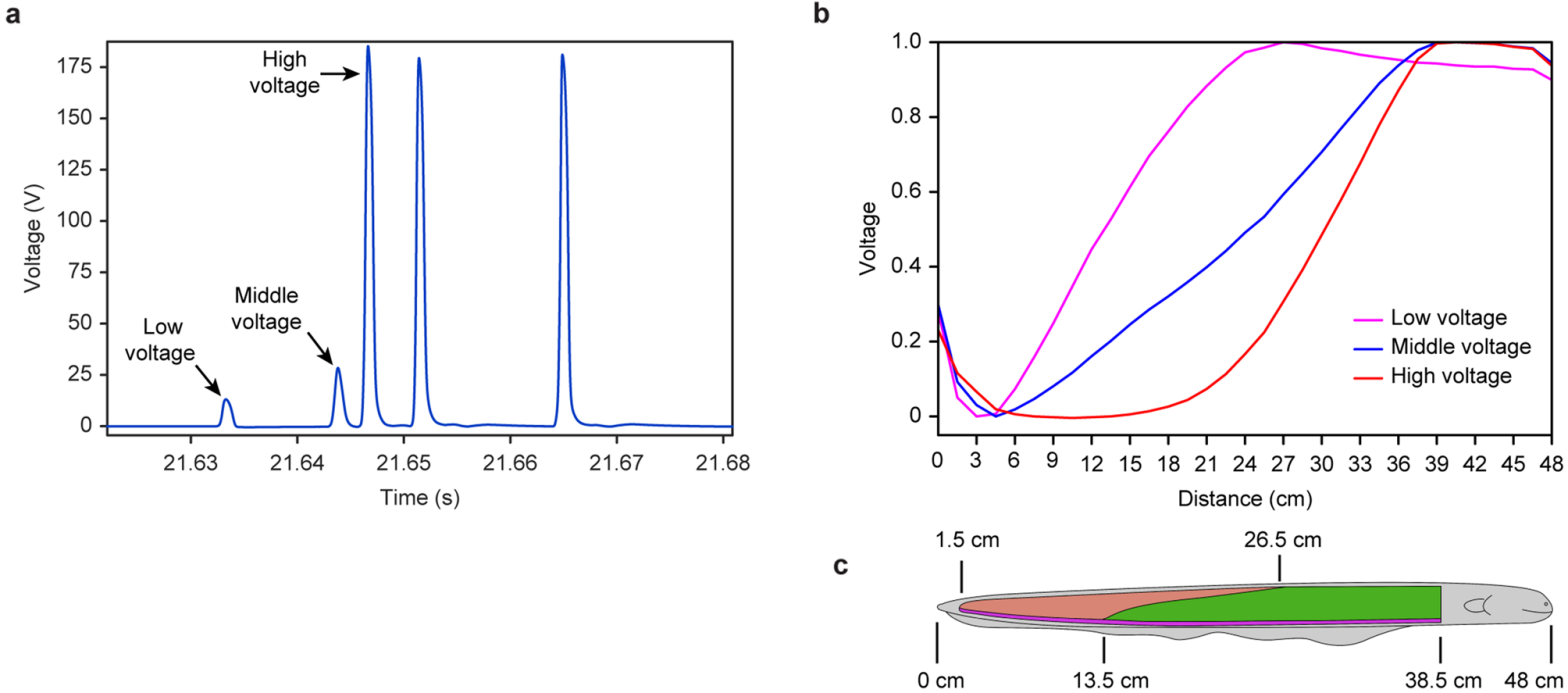
The typical scenario of electric eel’s discharge. (**a**) Comparing the three forms EOD time-domain waveform, which is an enlarged view of the EOD 5 in Fig. 2a. (**b**) Comparison of the three forms EOD voltage space distribution along the body of the 48 cm long electric eel (the normalized waveform refers to the voltage peak value of every EOD). (**c**) The 48 cm long electric eel and its three electric organs.

Although the middle-voltage EODs were easily recorded, and frequently generated, they have never previously been treated as an independent EOD type. According to the description above, we use the joint spatiotemporal analysis method: we use the EOD waveform voltage amplitude (time domain) as shown in Fig. 5a with the voltage space distribution (space domain) along the electric eel’s body as shown in Fig. 5b as the judge standard, confirmed that the middle-voltage pulse is a new form of independent EOD. Compared these three different EOD voltage space distributions with the three electric organ positions, as shown in Fig. 5b,c, and based on the fact that no evidence has arisen until now that electric eels can control part of their electric organs to carry out discharge, we believe that every form of EOD is generated by one electric organ independently. We can use these three basic EODs combination to explain irregular EODs waveform as hybrid EODs.

The amplitude difference between the middle-voltage EOD and the low-voltage EOD is not as apparent as the difference between the high-voltage EOD and the low-voltage EOD, as shown in Fig. 5a. Some hybrid EODs also exist, as Fig. 4a shows, which makes the question complicated. For this reason, the middle-voltage EOD has been overlooked since 1937. It has always been mixed with hybrid EODs and has been treated as an “over discharged” low-voltage EOD or an “unsuccessful” high-voltage EOD.

The middle-voltage EOD always occurs once between a low-voltage EOD and a high-voltage EOD. Figure 5a shows a typical scenario of electric eel discharge with time: The first pulse is the low-voltage discharge of Sachs’ organ at 21.6333 s, followed by the middle-voltage discharge of Hunter’s organ 10.5 ms later, and finally the high-voltage discharge of the main organ 2.9 ms later. Figure 3a also depicts a typical scenario of electric eel discharge. Supplementary Movie 5 demonstrates the changing process of this typical scenario of electric eel discharge. Sometimes no high-voltage EOD occurs, as shown by EOD 3 in Fig. 2a. The middle-voltage EOD occurs only one time following a low-voltage EOD. We never observe the middle-voltage EOD to occur repetitively and independently, as does the low-voltage EOD and high-voltage EOD. There are very few chances the middle-voltage EOD occur after a high-voltage EOD: we found only one time it occurred after a high-voltage EOD until now.

For these three basic EOD voltage amplitudes, based on the 45 s data of the 48 cm eel, the minimum value of the low-voltage EOD is 22.8 V, the maximum value of the low-voltage EOD is 25.8 V, and the difference is approximately 12%. The minimum value of the middle-voltage EOD is 38.5 V, the maximum value of the middle-voltage EOD is 56.5 V, and the difference is approximately 32%. The minimum value of the high-voltage EOD is 210 V, the maximum value of the high-voltage EOD is 279 V, and the difference is approximately 33%. The hybrid EODs voltage change from 52.9 to 212.2 V. The minimum value of the middle-voltage EOD is approximately 1.5 times the maximum value of the low-voltage EOD, and the minimum value of the high-voltage EOD is approximately 2.4 times the maximum value of the middle-voltage EOD.

When checking all the data in Fig. 2a, we found a total of 86 low-voltage EOD pulses, 15 middle-voltage EOD pulses, 64 high-voltage EOD pulses and 13 hybrid EOD pulses in the 45 s waveform. This outcome means that the middle-voltage EOD also occurs frequently. Among all 15 middle-voltage EODs, 14 were followed by high-voltage EOD, and only one was not followed by high-voltage EOD. For the high-voltage EOD, 14 followed the middle-voltage EOD, 2 followed the low-voltage EOD directly, and the others followed the high-voltage EOD or hybrid EOD. All the 13 hybrid EODs were generated by the conflict of the middle-voltage EOD and the high-voltage EOD.

We have also recoded the discharge of a 22 cm eel, a 42 cm eel and a 53 cm eel, all the data are summarized in Table 1.

**Table 1:** The EOD recording of 4 different length eels.

	22 cm eel, 40 s data	42 cm eel, 14 s data	48 cm eel, 45 s data	53 cm eel, 49 s data
Low-voltage EODs	52	76	86	32
Middle-voltage EODs	4	12	15	17
High-voltage EODs	7	49	64	41
Hybrid EODs	2	7	13	2
A	4	12	14	17
B	0	0	1	0
C	1	0	2	0

(a) Row A: Means the number of the middle-voltage EOD which followed the low-voltage EOD and was followed by high-voltage EOD; (b) Row B: means the number of the middle-voltage EOD followed the low-voltage EOD but was not followed by high-voltage EOD; (c) Row C: means the number of the high-voltage EOD which followed the low-voltage EOD directly without middle-voltage EOD.

These results show that the middle-voltage EODs are common among all these *Electrophorus* *electricus*. These results also show if an eel wants to launch a high-voltage EOD series after low-voltage EOD electrolocation, a 90% possibility that it will launch a middle voltage before the high-voltage EOD exists, and there is some possibility that the high-voltage EOD will conflict with the middle-voltage EOD. However, all of our data were recorded in an unnatural environment. The electric eel was placed in a narrow tank where it could not swim and move. We used a glass rod to touch its head, which threatened it.

Catania^16^ reported electric eel use pairs and triplet high-voltage EODs in complicated environments to stimulate hidden prey uncontrolled movement, which can enable eels find hidden living prey. Is middle-voltage EOD used to stimulate hidden prey? We do not believe so: (1) The amplitude of the middle voltage is 38.5–56.5 V in our experiment, and this voltage will attenuate greatly in deep water and at some distance. (2) The middle-voltage EOD occurs only one time after low-voltage EOD and before high-voltage EOD, and its duration is less than 2 ms. Thus, the amplitude and quantity are not sufficient to stimulate the prey to respond. Middle-voltage EOD occurs only once after a low-voltage EOD and before a high-voltage EOD, where it works as a “bridge” to “connect” the low-voltage EOD and high-voltage EOD. Even though sometimes no middle-voltage EOD exists between the low-voltage EOD and high-voltage EOD, we suppose that the middle-voltage EOD is used for coordinating the low-voltage EOD and high-voltage EOD, adjusting the electric eel body internal environment, such as balancing the charge inside. If used for any external function such as hunting or electrolocation, no reason exists for it to occur only one time and for less than 2 ms. More research is needed to reveal the function of middle-voltage EOD and the reason why electric eels evolved Hunter’s organ.

## Methods

### Animals

The experiments were performed in accordance with the Guide for the Care and Use of Laboratory Animals of the National Research Council. The experimental protocol was approved by the animal care and use committee of North China Electric Power University. *Electrophorus* *electricus* of 48 cm in length was purchased from a commercial fish supplier and housed in an 80 × 40 × 40 cm (L × W × H) glass tank with fresh water, and the water temperature was maintained between 20 and 30 °C. The eel was fed fish.

### Recordings of the EOD

To record the EOD, the electric eel was transferred to a 50 × 32 × 35 cm (L × W × H) customized Plexiglas aquarium. The EOD was recorded by 33 nickel-titanium alloy electrodes (0.5 mm in diameter, 10 cm in length) in the water, connected at their exposed tips to wire leads connected to the voltage divide circuit. We used a 100 K ohm/3.3 K ohm resistor serially connected to divide the electric eel EOD signal into ± 10 V spans. The output of the voltage divide circuit was connected to the input of the AD converter. We used two USB3103 AD converters, which have 16 channels each, and made them work synchronously to record 32 channel signals. Every channel has a 16-bit accuracy with a sample rate of 31.25 ksps. The 33 electrodes, voltage divider circuit, and AD converter module were all mounted on a 48 × 36 cm acrylic board. This board was used to push the electric eel to one side of the glass tank to ensure that all the electrodes were connected to its body well. Fresh water was added to the glass tank to a depth of 4 cm to ensure that every electrode recorded some voltage. The water temperature was 25–26 °C. The conductivity was 250 μS/cm. A glass rod was used to touch the electric eel’s head to evoke discharge, and all the sampling data were transferred to a Lenovo P50s laptop by a USB cable.

## Supplementary information

Supplementary Movie 1.

Supplementary Movie 2.

Supplementary Movie 3.

Supplementary Movie 4.

Supplementary Movie 5.

## Data Availability

The data that support the findings of this study are available from the authors upon reasonable request.
